# Polyneuropathy as Novel Initial Manifestation in a Case of “Nonsecretory” POEMS Syndrome with Sjögren's Syndrome

**DOI:** 10.1155/2017/1276759

**Published:** 2017-01-04

**Authors:** Minrui Liang, Zhixing Jiang, Zhiguang Lin, Bobin Chen, Hejian Zou, Weiguo Wan, Jun Liu

**Affiliations:** ^1^Division of Rheumatology, Huashan Hospital, Shanghai, China; ^2^Institute of Rheumatology, Immunology and Allergy, Fudan University, Shanghai 200040, China; ^3^Department of Hematology, Huashan Hospital, Fudan University, Shanghai 200040, China; ^4^Nursing Department, Huashan Hospital, Fudan University, Shanghai 200040, China

## Abstract

POEMS syndrome (polyneuropathy, organomegaly, endocrinopathy, monoclonal gammopathy, and skin changes) is a paraneoplastic syndrome driven by plasma cell dyscrasias. We report a patient with novel initial manifestation of polyneuropathy, which was considered due to Sjögren's syndrome but with poor response to methylprednisolone (120 mg/d) and intravenous immunoglobulin (IVIg). Further investigation by imaging tests and following biopsy eventually confirmed the diagnosis of POEMS syndrome secondary to solitary plasmocytoma. To our knowledge, this is the first reported case of POEMS syndrome with Sjögren's syndrome occurring in the absence of a peripheral monoclonal gammopathy, highlighting the diagnostic challenges posed by this disease and reviewing the diagnostic role of (18) F-FDG PET/CT in POEMS syndrome.

## 1. Introduction

POEMS syndrome is a rare paraneoplastic syndrome driven by neoplastic plasma cells. Diagnosis is often delayed as the syndrome is rare and can be mistaken for other diseases. The diagnosis of POEMS syndrome is confirmed in the presence of two mandatory major criteria (polyneuropathy and clonal plasma cell disorder), one of three other major criteria (sclerotic bone lesions, elevated VEGF, or Castleman disease), and at least one minor criterion [1]. Here, we present a case of POEMS syndrome complicated with Sjögren's syndrome (SjS), with significant initial manifestation of polyneuropathy but without a peripheral monoclonal gammopathy. In this case, making the diagnosis can be challenging, but radiographic examinations and biopsy analysis can help differentiate this syndrome from other conditions. Herein, this paper will review the diagnostic role of (18) F-FDG PET/CT in POEMS syndrome.

## 2. Case Report

A 58-year-old male was admitted to our center with a 6-month history of progressive numbness and weakness of extremities, complicated with dyspnea and repeated low fever. By a careful review of systems, positive results of eyes and arthralgia of the both knee joints were found. Inspection of his extremities showed noticeable hyperpigmentation. Physical examination showed decreased breath sounds. Neurological examination revealed predominant lower extremity weakness; the motor power in the upper extremities was MRC grade 4+, while the motor power in the lower extremities was MRC grade 4. The sensations of touch, pain, and vibration were decreased by 70% in the distal part of the extremities, although the position sense was intact. The patient's deep tendon reflexes were decreased. The cerebellar function test and the Romberg test showed no abnormalities. Neurophysiological evaluation revealed a sensory-motor demyelinating polyneuropathy. An ophthalmologic examination showed xerophthalmia and papilledema.

Computed tomography (CT) showed bilateral pleural effusions. Ultrasonography showed splenomegaly and subaxile lymphadenectasis. Besides, echocardiography and electrocardiogram findings were normal. Pulmonary function test indicated restrictive lung disease and reduced diffusing capacity of the lung for carbon dioxide (DLCO). Major laboratory results were shown in Table 1. The spinal fluid demonstrated markedly elevated protein level (1.13 g/L, normal range: 0.15–0.45 g/L). Pleural effusion analysis was normal, and no malignant cell was detected by a cytology exam. No infection evidence was found following repeated cultures of blood, pleural effusion, and cerebrospinal fluid. T-spot, procalcitonin, galactomannan (GM) antigen detection, or latex agglutination test was negative. Extensive immunologic testing was unremarkable. Bence Jones protein test result was negative. Repeated serum and urine immunofixation revealed no monoclonal gammopathy. There was no elevation of free kappa and lambda light chain in serum or urine, also with the normal light chain ratio (Table 1). Thyroid function tests revealed hypothyroidism (Table 1); however, the antibodies against thyroglobulin, thyroid peroxidase, or thyroid stimulating hormone (TSH) receptor were all negative, and the thyroid ultrasound showed normal results. Salivary gland scintigraphy with technetium revealed the reduction of radiotracer uptake and intake in gland. Biopsy of a minor labial gland showed typical lymphocytic infiltrates. Therefore, a diagnosis of primary Sjögren's syndrome (SjS) was confirmed following the positive findings including dry eyes, positive salivary scintigraphy, ocular staining score ≧3, and histopathological evidences (focus score, ≧1 focus/4 mm^2^), according to 2012 American College of Rheumatology (ACR) classification criteria for Sjögren's syndrome [2]. The patient's thyroid function returned to be normal after treating with Levothyroxine. Considering that his polyneuropathy was due to SjS, he was treated with methylprednisolone (120 mg/d) and IVIg for 3 days, and the dose of methylprednisolone was tapered to 60 mg/d. His temperature was back to normal then, but his paresthesia and weakness in extremities were not relieved remarkably.

**Figure 1:**
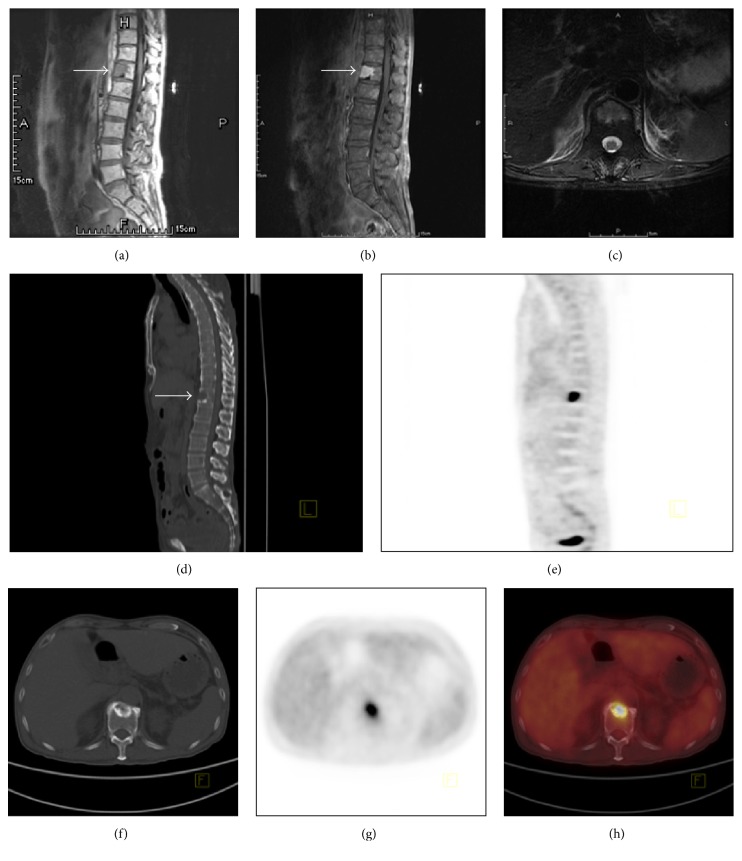
Images of the bone lesion detected by MRI and (18) F-FDG PET/CT. (a)~(c): Short-tau inversion-recovery (STIR) sequence in MRI shows a bone lesion in T12 vertebra, with T1-hypointense and T2-hyperintense signal. (a) Sagittal TI STIR image. (b) Sagittal T2 STIR image. (c) Transaxial T2 STIR image. (d)~(h): (18) F-FDG PET/CT image shows an osteosclerotic lesion, with hypermetabolism in T12 vertebra. (d) Sagittal CT image. (e) Sagittal PET image. (f) Transaxial CT image with a significant sclerotic ring around the bone lesion. (g) Transaxial PET image with an average SUV value of 12.4. (h) Fused PET/CT image.

**Figure 2:**
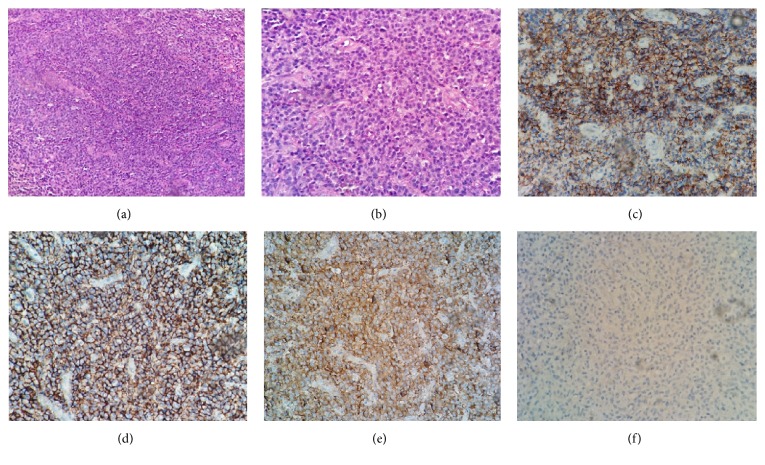
Histological analysis of the bone lesion in the body of T12. (a) The micrograph shows the accumulation of plasma cells with the destruction of trabecular bones [hematoxylin and eosin (H&E) staining; magnification, ×200]. (b) Higher magnification of the image in (a) (H&E staining; magnification, ×400). Immunohistochemical staining shows positive expression of (c) CD38 (++), (d) CD138 (++), (e) lambda (++), and (f) kappa (−) (magnification, ×400).

Magnetic resonance imaging (MRI) of the spine revealed a bone lesion in the body of T12, measuring about 21.1 mm (Figure 1). (18) F-FDG PET/CT showed an isolated osteosclerotic bone lesion in T12 with the increased FDG uptake (the average SUV is 12.4) (Figure 1). Histological examinations of the bone lesion biopsy showed plasmacytoma with predominance of lambda light chain expression in the plasma cell aggregates (Figure 2). Additionally, the immunohistochemical examination revealed CD38 (++), CD138 (++), CD56 (−), CD117 (±), CD20 (−), Cyclin D1 (±), MUM-1 (+), kappa (−), lambda (++), Bcl-2 (±), Ki-67 (<1% positive), EMA (+), and CD19 (−). Cytological and histological examination of the bone marrow showed no proliferation of plasma cells. Serum VEGF level was highly elevated (>800 pg/mL, normal range: 0~142). POEMS syndrome secondary to solitary plasmocytoma was diagnosed based on polyneuropathy, splenomegaly, hypogonadism, plasmacytoma, osteosclerotic bone lesion, VEGF elevation, pleural effusion, and skin changes, which were consistent with the proposed diagnostic criteria [1]. The patient died of rapid disease progression of polyserositis before starting radiotherapy.

## 3. Discussion

The challenges in diagnosing this case lied in (1) the overlapping manifestation of polyneuropathy in POEMS syndrome and SjS and (2) the undetectable monoclonal gammopathy. POEMS syndrome is a rare variant of plasma cell neoplasm. Nonsecretory plasmacytoma is also a rare form of plasmacytoma characterized by the absence of detectable monoclonal gammopathy in serum and urine. Misdiagnosing or mislabeling the patients with plasmacytoma results in the delay of their urgent treatments. In this case, comprehensive imaging studies and histopathologic confirmation are necessary.

Firstly, we will analyze and compare the peripheral neuropathy patterns in POEMS syndrome and SjS. Demyelinating polyneuropathy is a typical characteristic of POEMS syndrome; also it is a mandatory major criterion for diagnosing POEMS. Endothelial injury is a leading cause in the progression of polyneuropathy in POEMS syndrome, and the overproduction of VEGF enhances microvascular permeability and subsequent endoneurial edema [8, 9]. No direct correlation has been observed between the levels of monoclonal gammopathy and the severity of neuropathy. Although our patient did not have a demonstrable paraprotein, he had a proven plasmacytoma with lambda light chain clonality, which has been reported in previous literature regarding POEMS [10, 11]. Based on the extent of the plasma cell infiltration, targeting therapy at the underlying clonal plasma cell could improve symptoms and even cure the patients [1]. In this case, since the patient had a solitary plasmacytoma without clonal plasma cells found in bone marrow biopsy, radiation is the recommended therapy [1]; however, he had no chance for radiotherapy.

The prevalence of SjS associated peripheral neuropathy is reported in recent studies, ranging from 2% to over 60% [12, 13]. The clinical spectrum of peripheral neurologic involvement in SjS is wide, and all segments of peripheral nervous system can be involved, with sensory neuropathies being the most common [12, 13]. Cranial neuropathy, multiple mononeuropathy, demyelinating polyneuropathy, and autonomic neuropathy are less frequently encountered types [12, 13]. Autoimmunity induced vasculitis and autoantibodies are thought to be the contributors in the pathogenesis of SjS associated polyneuropathies [14]. Demyelinating polyneuropathies have been reported in some cases [12, 13, 15]. This type of neuropathy is usually much severer and typically presents with proximal and distal weakness and sensory deficits with subacute onset. Cerebrospinal fluid studies reveal elevated protein level with normal cell count, while the neurophysiological evaluation shows the typical demyelination changes [16]. Prompt treatment with steroids and IVIg is reported to be an effective therapeutic intervention for this type of neuropathy [16, 17].

Secondly, we will review the diagnostic role of (18) F-FDG PET/CT in POEMS syndrome. In this case, MRI detected the bone lesion but not revealing the typical sclerotic property; CT revealed an osteosclerotic lesion; (18) F-FDG PET/CT confirmed its superiority in defining both skeletal lesions and the activity of the neoplastic process. We herein conclude that the different manifestations of the disease imply the necessity of a complex evaluation of imaging methods in mutual concordance. (18) F-FDG PET/CT emerges as the most contributive method for evaluating both the extent and the activity of this disease (Table 2).

POEMS syndrome presents with a constellation of signs and symptoms that may lead a clinician to a multitude of other possible diagnosis. There exist overlapping clinical signs between POEMS and SjS, and confirmed diagnosis can be made based on detailed clinical history, physical examination, imaging tests, and biopsy analysis.

## Figures and Tables

**Table 1:** Laboratory results.

Variable	Value (normal range)
Hemoglobin, g/L	164 (130–175)
White blood cell count, ×10^9^/L	6.43 (3.5–9.5)
Neutrophil, ×10^9^/L	4.12 (1.8–6.3)
Platelet count, ×10^9^/L	163 (125–350)
Creatinine, serum (SCr), umol/L	90 (59–104)
Albumin, g/L	35.5 (40–55)
Alanine transaminase (ALT), U/L	29 (9–50)
Aspartate aminotransferase (AST), U/L	24 (15–40)
Prothrombin time (PT), sec	11.7 (9.6–12.2)
Activated partial thromboplastin time (APTT), sec	27.4 (20.3–32.3)
D-Dimer, FEU mg/L	0.60 (<0.55)
Immunoglobulin (Ig) G, g/L	23.4 (7–16)
IgM, g/L	0.58 (0.4–2.30)
IgA, g/L	0.92 (0.7–4.0)
Serum kappa, mg/L	3.31 (1.7–3.7)
Serum lambda, mg/L	1.78 (0.90–2.10)
Serum kappa/lambda	1.85 (1.35–2.65)
Urine kappa, mg/L	2.10 (<4.10)
Urine lambda, mg/L	1.69 (<7.50)
Urine kappa/lambda	1.24 (0.70–4.5)
Creatine phosphokinase (CPK), U/L	13 (39–308)
Probrain natriuretic peptide (BNP), pg/mL	1393 (0–210)
Lactate dehydrogenase (LDH), U/L	181 (135–225)
Testosterone, nmol/L	4.21 (6.68–25.70)
Dehydroepiandrosterone, umol/L	0.27 (1.91–13.4)
Thyroid stimulating hormone (TSH), mIU/L	11.99 (0.34–5.60)
Free triiodothyronine (FT3), pmol/L	2.40 (3.80–6.00)
Free thyroxine (FT4), pmol/L	8.67 (7.86–21.10)
Free cortisol in urine, ug/24 h	412.17 (30.15–129.13)
Serum calcium, mmol/L	2.16 (2.15–2.50)
Beta 2-microglobulin, mg/L	4.48 (0.9–3.10)
Vascular endothelial growth factor (VEGF), pg/mL	>800 (0–142)
Cerebrospinal fluid (CSF) glucose, mmol/L	4.28 (2.22–3.89)
Cerebrospinal fluid (CSF) protein, g/L	1.13 (0.15–0.45)
Cerebrospinal fluid (CSF) chloridum, mmol/L	126 (120–132)
Cerebrospinal fluid (CSF) Pandy's test	2+
Cerebrospinal fluid (CSF) white blood cell, 10^6^/L	0 (0–8)
Cerebrospinal fluid (CSF) red blood cell, 10^6^/L	2

**Table 2:** Clinical characteristics of POEMS cases in published reports.

Author and reference	Year of study	Study design	Number of POEMS patients	Technical procedure used	Other diagnostic images	Primary outcome
Glazebrook et al. [3]	2015	Retrospective clinical study	24	(18) F-FDG PET/CT	X-ray and CT	Diagnosis
Royer et al. [4]	2013	Retrospective clinical study	12	(18) F-FDG PET/CT	X-ray and CT	Diagnosis (11/12 patients have positive findings); evaluation of clinical response to therapies
Minarik et al. [5]	2012	Case report	3	(18) F-FDG PET/CT	X-ray, CT, technetium scintigraphy, MRI, and angiography	Diagnosis
Montoriol et al. [6]	2011	Case report	2	(18) F-FDG PET/CT	X-ray, CT, MRI, and technetium scintigraphy	Diagnosis
Albertí et al. [7]	2010	Case report	4	(18) F-FDG PET/CT	X-ray, CT, and technetium scintigraphy	Diagnosis
